# Talks about Food

**Published:** 1892-03-19

**Authors:** 


### Talks about Food.

IV.—SUCKING THE HONEY FROM THE BEE.

One cannot but be amused at the ardent fanaticism and the "gush" of the Vegetarian. Persons coming fresh to his writings must rub their eyes, and ask themselves whether they can by any possibility have mistaken a religious tract for a vegetarian leaflet. And, indeed, one might often hand over such a leaflet to a person asking for a tract, without fear of detection in the fraud, so full will it be of phrases about "the redemption of the world," "the gospel of the living God," and "divine pre-ordinance." We have even heard of an ardent vegetarian being introduced by a friend as "another Christ"! And as to the adulation heaped upon the orange, Matthew [Arnold's humorous remarks about the ideal world of the Radical, where we all should blissfully marry our deceased wife's sister, might with a little variation apply to the vegetarian's paradise—in which all disease, all ill-temper, and almost all sin, should flee away at sight of that magic "golden pill," the divinely appointed means for "restraining the brutality, and strengthening the mentality, of the population"!

None the less, we owe the vegetarians a debt of gratitude. It has been well said that "every true scientist is under deep obligations to the vegetarians for performing what is a valuable dietetic experiment, important both physiologically and economically." And more than this. There can be no doubt that the vegetarians have a considerable amount of reason on their side, and that they are doing good service to the world by showing that animal food is not so all-important as many think it, and by bringing before our notice various foods which have been hitherto neglected, or looked upon as serving only for the gratification of our taste. To the well-to-do their teaching is useful, in that it suggests to us a more healthful diet, and teaches us how to make this palatable; and to the poor it is still more so, for a good vegetarian dish adds to its wholesomeness strength, and to its strength economy. Valuable instruction is afforded to the people by the "specimen suppers" which are given now and again, the *menu* consisting solely of vegetarian dishes; and better still would be practical demonstrations, in which the dishes should be prepared in presence of, or by the guests (probably some such are given), before being eaten.

Yes, the vegetarian bee is a most industrious and praiseworthy insect; and if we do not care for all his products, or at least for all the uses to which he puts them, he yet has plenty of good honey to offer us, and we shall be doing very foolishly if we refuse to take it.

Whether or no flesh-eating has a tendency to make men fierce, is a question on which we will not offer an opinion. Lord Byron thought it had, and his friend Moore relates how one day, when he (Moore) was devouring a mutton chop, he found Byron's eyes fixed curiously upon his face, and at last was asked, "Don't you find that a meal like that makes you feel very ferocious?" And Liebig says that "it is essentially their food which makes carnivorous animals in general bolder and more combative than the herbivora." It is not exactly pleasant to think of ourselves as "fierce"; let us hope that the word is only applicable to us in the sense in which it is used by the East Anglian peasantry, with whom a "fierce child" is only a strong and lively one. But, after all, boldness and combativeness are very necessary qualities if we are to make our way in the world. If we are not mistaken, all the leading nations, past and present, have been flesh-eaters: and those who live entirely, or almost entirely, on rice or roots are practically of no account. Moreover, we must have a certain amount of nitrogenous matter, and the easiest way to take it is in animal food. It cannot be wise to tax our digestive organs with an undue quantity of food—as we are practically compelled to do if it is not of the right quality—in order to obtain the proper amount of some necessary element.

Peas, beans, and lentils, by the way, are rich in nitrogenous matter, and may well be tried by persons having fairly strong digestions. But those who think of making a change in their diet should take great care that they do it sensibly and scientifically. To insist that what is generally found wholesome for the human race must be wholesome for each individual is a mistake which may lead to serious consequences, especially when, say, a young family of children has to be provided for. The abundance of fruit on which vegetarians insist, and which may have an excellent effect on one person, may make another person ill; and while one of us has a cow-like power of digesting succulent vegetables, another, with a weak digestion, will be made wretched by trying to follow his example. Still more serious is the mistake of arbitrarily selecting a diet from which some necessary element of nutrition is altogether absent. The editor of the *Vegetarian* himself mentions two cases in which children, being weaned from their mother's milk, were fed entirely on a dietary of fruit and grain foods, the result being that though the children appeared functionally healthy, they grew not at all, and seemed to have no muscular energy, only recovering when a liberal admixture of "pulse" was tried. Here, a little knowledge as to the composition of fruits and grains, and the various elements required by the human frame, would have averted the mischief; and the same may be said of those sad cases in which children have been practically starved to death by being fed on starch (*i.e.*, arrowroot) alone. To give one more instance of the way in which people will sometimes catch up a certain truth and carry it to a highly unscientific length, we have known a lady (not a vegetarian) who, believing—very rightly—that over-eating was a prevalent evil, leaped out of the frying-pan into the fire, and so stinted her family of growing girls, that they went about looking dull and miserable, and quite failed to conceal their joy, big girls though they were, when a sympathising visitor added some simple meat pies to their evening meal.

We have laughed a little at the adoration of the orange, true; but there can be no doubt that if fruit took a more prominent part in our dietary than it does in most households, it would be all the better for those households—and all the worse for the chemists. People banish from their tables Nature's purifier of the blood, and then fly to physic to remedy the evil which they have caused by their own foolishness. And as all drugs derange gastric digestion more or less, it is folly to use them except when we must—to lose sight of the truth that prevention is far better than cure.

The vegetarian, then, is by no means to be despised. He has plenty to say that is well worth our attention; and if we wish to do good to ourselves and our fellow creatures, we might do far worse than study and give away some of his less fanatical books and leaflets—such, for example, as Dr. Nichols' "How to Live Well on Sixpence a Day," or a book of simple vegetarian recipes.

